# SREBP-driven lipogenesis coupled with ferroptosis resistance as a therapeutic target in PTEN-loss extrahepatic cholangiocarcinoma

**DOI:** 10.1016/j.jhepr.2026.102015

**Published:** 2026-08-29

**Authors:** Yuki Hayata, Yuki Matsushita, Mina Tempaku, Shun Nakagawa, Tomoharu Yamada, Satoshi Kawamura, Kiyora Izuoka, Genki Kimura, Yutaro Tachi, Hideaki Tanaka, Akiko Eguchi, Naoto Fujiwara, Hidemasa Goto, Hayato Nakagawa

**Affiliations:** 1 Department of Gastroenterology and Hepatology, Mie University Graduate School of Medicine, Tsu, Japan; 2 Department of Gastroenterology, The University of Tokyo, Tokyo, Japan; 3 Department of Histology and Cell Biology, Mie University Graduate School of Medicine, Tsu, Japan

**Keywords:** Cholangiocarcinoma, PTEN, SREBP, Lipogenesis, Ferroptosis

## Abstract

**Background & Aims:**

Extrahepatic cholangiocarcinoma (eCCA) is an aggressive biliary tract cancer (BTC) with limited therapeutic options and actionable genetic alterations. Although PTEN loss and PI3K pathway activation are common in eCCA, their roles in disease pathogenesis and therapeutic vulnerability remain unclear. Therefore, we aimed to establish clinically relevant mouse models of PTEN loss-driven eCCA and identify novel therapeutic strategies.

**Methods:**

To investigate eCCA development and the underlying mechanisms, we generated *CK19-Cre*^*ERT*^-based genetically engineered mouse models with biliary epithelial cell-specific PTEN deletion combined with either TGFβR2 loss or oncogenic Kras activation.

**Results:**

PTEN loss alone was insufficient for tumorigenesis, whereas combined deletion of PTEN and TGFβR2 induced periductal infiltrating-type eCCA originating from the peribiliary glands. In contrast, PTEN loss and oncogenic Kras activation predominantly generated intraductal-growing tumors with broader biliary involvement. These included intrahepatic cholangiocarcinoma (iCCA) and gallbladder cancer (GBC). RNA sequencing revealed that SCAP/SREBP-mediated lipogenesis was consistently activated in the PTEN loss-driven eCCA models. Genetic ablation of SCAP markedly suppressed eCCA development (*p* <0.0001) but exacerbated iCCA, indicating the need for alternative therapeutic approaches. SREBP-driven lipogenesis increased the synthesis of polyunsaturated phospholipids and concomitantly induced GPX4, thereby conferring resistance to ferroptosis. GPX4 inhibition suppressed eCCA (*p* = 0.002) and GBC development without exacerbating iCCA. Consistently, PTEN-deficient human BTC cell lines, including CRISPR–Cas9-engineered models, exhibited increased vulnerability to GPX4 inhibition. Furthermore, clinical eCCA samples (n = 182) confirmed the coordinated activation of the PI3K, SREBP, and ferroptosis pathways.

**Conclusions:**

Distinct oncogenic alterations shape the histological and anatomical diversity of BTC. PTEN loss-driven eCCA critically depends on SCAP/SREBP-mediated lipid reprogramming and GPX4-dependent ferroptosis resistance, indicating the therapeutic potential of ferroptosis induction.

**Impact and implications:**

This study provides a scientific rationale for the stratification of extrahepatic cholangiocarcinoma (eCCA) based on oncogenic context. Our findings demonstrate that PTEN loss is associated with shared lipid metabolic features, whereas additional genetic alterations determine histological growth patterns and anatomical distribution. We identified ferroptosis resistance as a key downstream feature of PTEN-deficient eCCA. Although further validation is required, these findings provide a theoretical basis for further exploring ferroptosis-based therapeutic approaches for genetically defined subsets of biliary tract cancer.

## Highlights

• New mouse models recapitulate the histological diversity of PTEN-deficient BTCs.
• SCAP/SREBP-driven lipogenesis is essential for PTEN-deficient eCCA.
• SCAP deletion exerts opposite effects in eCCA and peripheral iCCA.
• GPX4 inhibition exploits ferroptosis vulnerability in PTEN-deficient eCCA.

## Graphical abstract

**Figure:**
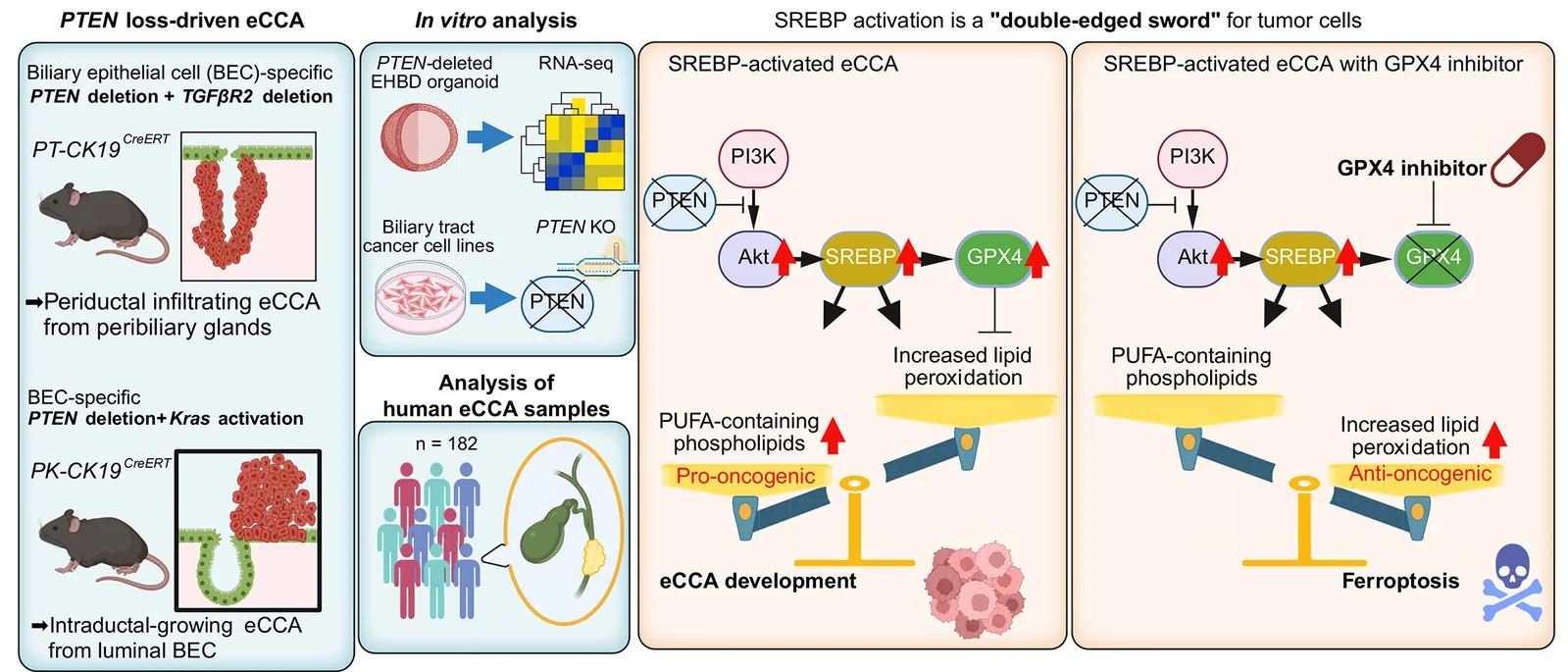

## Introduction

Biliary tract cancers (BTCs) are a group of malignancies found in the epithelial lining of the biliary system. They are anatomically classified as intrahepatic cholangiocarcinoma (iCCA), perihilar cholangiocarcinoma (CCA), distal CCA, or gallbladder cancer (GBC).^1^^,^^2^ Perihilar and distal CCA are collectively referred to as extrahepatic CCA (eCCA). In addition to its anatomical location, CCA can be further classified by tumor morphology into mass-forming, periductal infiltrating, and intraductal-growing types.^3^ BTCs are aggressive malignancies with poor prognoses, especially in patients with unresectable advanced-stage disease.^4^ Recent genomic and molecular profiling studies have revealed substantial differences in the mutational landscapes and biological behaviors of BTC subtypes, indicating their distinct anatomical and embryological origins.^5^^,^^6^ For example, approximately 40% of iCCA cases harbor potentially actionable genetic alterations, such as *FGFR2* fusions. Thus, precision medicine based on genetic panel testing is increasingly applied in clinical practice.^7^ In contrast, eCCA and GBC, particularly eCCA, exhibit a few druggable mutations, highlighting the urgent need for novel therapeutic strategies.

The availability of suitable animal models facilitates the elucidation of carcinogenic mechanisms and the development of targeted therapies. Although various genetically engineered and chemically induced mouse models of iCCA have been established,[8], [9], [10], [11], [12] experimental models of eCCA remain extremely limited. The lack of reliable animal models has significantly hindered mechanistic investigations and preclinical testing of therapeutic approaches for eCCA. To address this limitation, we previously established a genetically engineered mouse model of eCCA by inducing biliary epithelial cell (BEC)-specific activation of oncogenic *Kras*^*G12D*^ in combination with the deletion of *Tgfbr2* and *Cdh1* in *CK19-Cre*^*ERT*^ mice (*KTC-CK19*^*CreERT*^ mice).^13^ This model accurately mimics the histological features of the periductal infiltrating type of human eCCA, particularly under chronic biliary inflammation; the loss of E-cadherin (*Cdh1*) induces epithelial injury and inflammation, which consequently promotes cholangiocarcinogenesis.

eCCA comprises various genetic subtypes. Thus, a single model cannot fully represent its molecular diversity. Activation of the PI3K pathway and inactivation of PTEN, a negative regulator of this pathway, are common recurrent alterations in both iCCA and eCCA.^5^^,^^6^ These alterations have been implicated in resistance to immune checkpoint inhibitors,^14^^,^^15^ which have emerged as promising therapeutic options for CCAs. Although PI3K and Akt inhibitors are currently under clinical development for BTC treatment, their therapeutic efficacy remains insufficient.^16^^,^^17^ In the present study, we aimed to facilitate the development of new therapeutic strategies by elucidating the mechanisms underlying carcinogenesis. To this end, we developed and characterized novel eCCA mouse models based on PTEN loss in BECs.

## Materials and methods

### Animal experiments

*Albumin-Cre*, *Lox-Stop-Lox(LSL)-Kras*^*G12D*^, and *Scap*^*flox/flox*^ mice were purchased from Jackson Laboratory. *CK19-Cre*^*ERT*^, *Tgfbr2*^*flox/flox*^, *Pten*^*flox/flox*^, and *Rosa26-LSL-LacZ* mice have been described previously (Bar Harbor, ME, USA).[18], [19], [20], [21] All strains except *Scap*^*flox/flox*^ mice were of the C57BL/6 genetic background, and *Scap*^*flox/flox*^ mice were backcrossed with the C57BL/6 strain at least 10 times.^22^ The genotypes and abbreviations of the genetically engineered mice used in this study are summarized in Table 1. All experiments were approved by the Ethics Committee for Animal Experimentation of the University of Tokyo and Mie University and were conducted in accordance with the Guidelines for the Care and Use of Laboratory Animals.

**Table 1:** Genotypes and abbreviations of the genetically engineered mice used in this study.

Mouse model	Genotype			
*P-CK19-Cre*^*ERT*^	*CK19-Cre*^*ERT*^	*Pten*^*flox/flox*^		
*PT-CK19-Cre*^*ERT*^	*CK19-Cre*^*ERT*^	*Pten*^*flox/flox*^	*Tgfbr2*^*flox/flox*^	
*PT-LacZ-CK19-Cre*^*ERT*^	*CK19-Cre*^*ERT*^	*Pten*^*flox/flox*^	*Tgfbr2*^*flox/flox*^	*Rosa26-LSL-LacZ*
*PK-CK19-Cre*^*ERT*^	*CK19-Cre*^*ERT*^	*Pten*^*flox/flox*^	*LSL-Kras*^*G12D*^	
*PTS-CK19-Cre*^*ERT*^	*CK19-Cre*^*ERT*^	*Pten*^*flox/flox*^	*Tgfbr2*^*flox/flox*^	*Scap*^*flox/flox*^
*PKS-CK19-Cre*^*ERT*^	*CK19-Cre*^*ERT*^	*Pten*^*flox/flox*^	*LSL-Kras*^*G12D*^	*Scap*^*flox/flox*^
*KTC-CK19-Cre*^*ERT*^	*CK19-Cre*^*ERT*^	*LSL-Kras*^*G12D*^	*Tgfbr2*^*flox/flox*^	*Cdh1*^*flox/flox*^
*PK-Alb*	*Albumin-Cre*	*Pten*^*flox/flox*^	*LSL-Kras*^*G12D*^	
*PKS-Alb*	*Albumin-Cre*	*Pten*^*flox/flox*^	*LSL-Kras*^*G12D*^	*Scap*^*flox/flox*^

### Immunoblotting and real-time PCR analyses

Immunoblotting and RNA extraction were performed as previously described.^23^ The relative amount of each mRNA was quantified using real-time PCR and normalized to *B2M* mRNA expression. The primer sequences used are listed in Table S1.

### Histological analyses

Liver and bile duct tissues were processed as previously described (see Supplementary Materials and methods)^13^^,^^24^ for H&E staining, immunostaining, and LacZ staining. *In situ* hybridization (ISH) was performed using the RNAscope 2.5 HD Reagent Kit RED (Advanced Cell Diagnostics, Newark, CA, USA).^25^ The mouse biliary tree was classified into three anatomical regions: (1) extrahepatic bile ducts (EHBDs); (2) large intrahepatic bile ducts (IHBDs), corresponding to the proximal IHBDs (approximately up to second-order branches); and (3) peripheral IHBDs. The maximum diameter of the EHBD was measured as previously described.^13^

### Cell line and organoid experiments

Gene targeting using the CRISPR–Cas9 system was performed as previously described.^26^^,^^27^ The EHBD organoids were cultured as previously described.^13^

### Analyses of human samples

Transcriptomic profiles of 182 human eCCA tissues were extracted from the NCBI Gene Expression Omnibus database (www.ncbi.nlm.nih.gov/geo, accession number GSE132305).^28^

### Statistical analyses

Statistical analyses were performed using Student’s *t* test or Tukey’s multiple comparison test, as appropriate. Data are expressed as the mean ± SEM. Survival comparisons were performed using Fisher’s exact test. Values of *p* <0.05 were considered statistically significant, as indicated by asterisks (∗) in the figures. Data analyses were performed using GraphPad Prism (version 10.0; GraphPad Software, Boston, MA, USA) and R statistical software (R Foundation for Statistical Computing, Vienna, Austria).

Additional details are provided in the Supplementary Materials and methods.

## Results

### Combined BEC-specific PTEN loss and TGFβR2 deletion drives eCCA development

We used *CK19-Cre*^*ERT*^ mice in which tamoxifen (TAM)-inducible Cre^ERT^ is knocked into the endogenous *CK19* locus to induce BEC-specific gene alteration.^18^ We previously reported that *CK19-Cre*^*ERT*^ mice are suitable for the efficient induction of gene recombination in BECs (∼40%) throughout the biliary tree, including the peribiliary glands (PBGs), and for the subsequent development of eCCA.^13^ In the present study, we crossed *CK19-Cre*^*ERT*^ mice with *Pten*^*flox/flox*^ mice to induce PTEN loss in BECs (*P-CK19*^*CreERT*^ mice). As in our previous study, TAM was administered for 3 consecutive days at 7–8 weeks of age. However, even after 90 days of observation, only mild hyperplasia of the surface BECs and PBGs was observed in the EHBD, and no apparent tumor formation was detected (Fig. 1A).

**Fig. 1:**
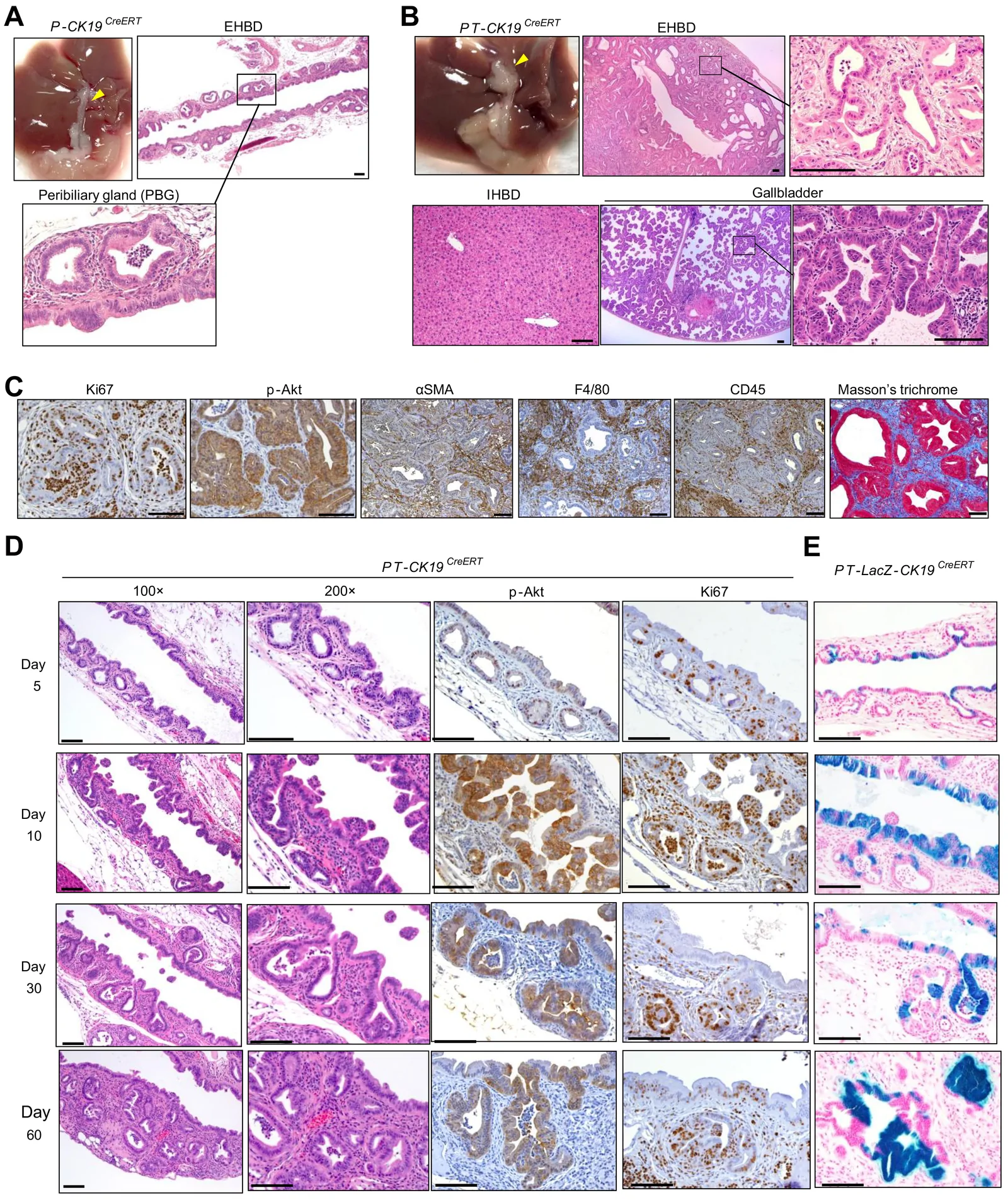
BEC-specific PTEN loss combined with TGFβR2 deletion drives eCCA development. (A,B) Representative macroscopic images of the EHBD and corresponding H&E-stained sections from *P-CK19*^*CreERT*^ mice (A) and from the EHBD, IHBD, and gallbladder of *PT-CK19*^*CreERT*^ mice (B) at 90 days after TAM administration. The magnified image in (A) shows dysplastic PBGs. (C) IHC staining for the indicated proteins and Masson’s trichrome staining of eCCA tissues from *PT-CK19*^*CreERT*^ mice. (D) H&E-stained sections and IHC staining for indicated proteins in the EHBD of *PT-CK19*^*CreERT*^ mice at the indicated time points after TAM administration (n = 6–7 mice per time point). (E) LacZ staining of the EHBD from *PT-LacZ-CK19*^*CreERT*^ mice at the indicated time points after TAM administration (n = 5 mice per time point). Scale bars, 100 μm. BEC, biliary epithelial cell; eCCA, extrahepatic cholangiocarcinoma; EHBD, extrahepatic bile duct; IHBD, intrahepatic bile duct; IHC, immunohistochemistry; PBG, peribiliary gland; TAM, tamoxifen.

These findings suggested that additional oncogenic events are required for tumorigenesis. TGFβ/SMAD signaling pathways are frequently mutated in human eCCA,^5^^,^^6^ and TGFβR2 deletion promotes eCCA development.^13^ Therefore, we further crossed *P-CK19*^*CreERT*^ mice with *Tgfbr2*^*flox/flox*^ mice to generate *Pten*/*Tgfbr2* double-knockout (KO) mice (*PT-CK19*^*CreERT*^ mice). BEC-specific deletion of TGFβR2 alone did not result in apparent histological abnormalities in the EHBD, as previously reported.^13^ In contrast, *PT-CK19*^*CreERT*^ mice showed a progressive decline in their general condition at approximately 90 days after TAM administration. This decline is occasionally characterized by jaundice. Therefore, the mice were euthanized for further analyses. *PT-CK19*^*CreERT*^ mice exhibited extrahepatic biliary wall thickening. Of these mice, 75% (15/20; males, n = 9/12; females, n = 6/8) developed macroscopically visible tumor lesions predominantly located between the perihilar region and the junction with the cystic duct (Fig. 1B). In contrast, no neoplastic changes were observed in IHBDs. Although hyperplastic changes were frequently observed in the gallbladder, overt GBC developed in only two mice (Fig. 1B). Histologically, most of the neoplastic lesions in EHBD were moderately differentiated adenocarcinomas with a glandular architecture (Fig. 1B). Immunostaining revealed high proliferative activity, as evidenced by frequent Ki67 positivity. Strong Akt phosphorylation, which was consistent with the loss of PTEN, was also observed (Fig. 1C). Similar to human eCCA, Masson’s trichrome staining revealed marked collagen deposition in the tumor stroma, along with abundant α-smooth muscle actin-expressing activated fibroblasts and infiltration of CD45-positive immune cells, including F4/80-positive macrophages (Fig. 1C). These findings indicate that eCCA arising in *PT-CK19*^*CreERT*^ mice closely recapitulates the tumor microenvironment of human eCCA.

We further analyzed the process of eCCA development in *PT-CK19*^*CreERT*^ mice. Ten days after TAM administration, papillary lesions were observed in the bile duct lumen. However, these lesions gradually disappeared by Day 30 (Fig. 1D). In contrast, PBGs exhibited proliferation and atypical changes that progressively expanded within the bile duct wall. Adenocarcinoma infiltration along the bile duct wall was evident by Day 60, displaying a periductal infiltrating pattern in all analyzed mice (n = 7/7, 100%) (Fig. 1D). Immunohistochemistry (IHC) revealed that p-Akt was abundantly expressed in luminal epithelial cells up to Day 10. However, its expression declined thereafter, whereas atypical PBG cells showed strong p-Akt positivity (Fig. 1D). Ki67 staining showed similar temporal and spatial patterns, supporting the shift in proliferative activity from luminal epithelial cells to PBG-derived cells (Fig. 1D). Because PBGs have recently been recognized as a BEC stem cell niche and a potential cell of origin for CCA,^25^^,^^29^^,^^30^ we performed lineage tracing by crossing *PT-CK19*^*CreERT*^ mice with *Rosa26-LSL-LacZ* mice (*PT-LacZ-CK19*^*CreERT*^). On Day 10, LacZ-positive cells were observed in both the luminal epithelia and PBGs. However, luminal positivity gradually diminished, whereas LacZ-positive PBG cells expanded and underwent malignant transformation (Fig. 1E). Together, these findings suggest that PBGs may serve as the cells of origin for eCCA in *PT-CK19*^*CreERT*^ mice, leading to the development of tumors with a periductal infiltrating growth pattern.

### Distinct oncogenic alterations shape BTC distribution and eCCA growth patterns

To further investigate the role of individual genetic alterations in extrahepatic cholangiocarcinogenesis, we generated mice with a BEC-specific PTEN deletion and Kras activation by crossing *P-CK19*^*CreERT*^ mice with *LSL-Kras*^*G12D*^ mice (*PK-CK19*^*CreERT*^ mice). This model was previously reported to develop both iCCA and eCCA.^8^ All *PK-CK19*^*CreERT*^ mice developed eCCA within 4 weeks of TAM administration (n = 18; 10 males and 8 females) (Fig. 2A). In contrast to *PT-CK19*^*CreERT*^ mice, GBC and iCCA were observed in all *PK-CK19*^*CreERT*^ mice; iCCA was predominantly detected in the large IHBDs (Fig. 2A).

**Fig. 2:**
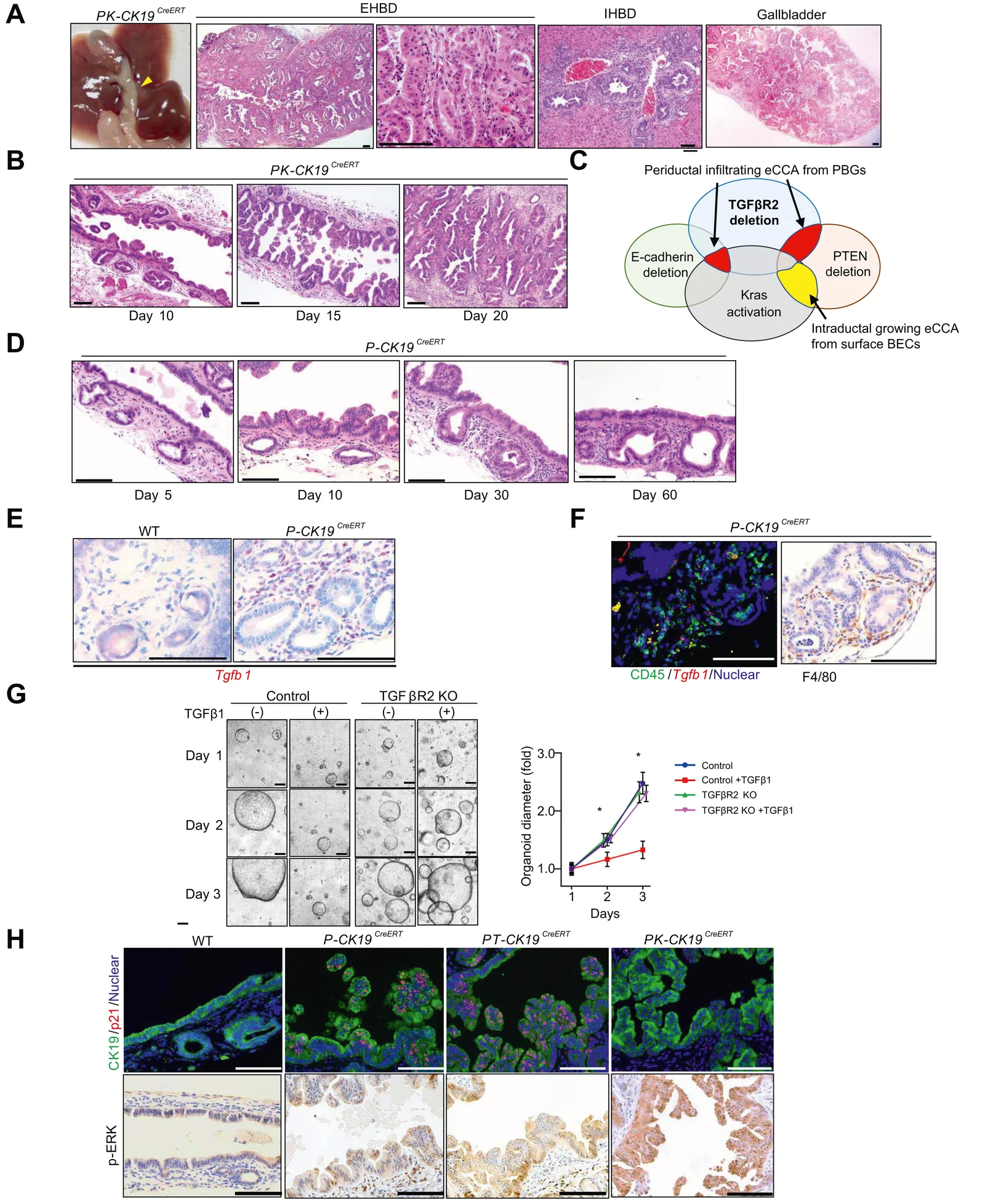
Distinct oncogenic alterations shape BTC distribution and eCCA growth patterns. (A) Representative macroscopic image of the EHBD and H&E-stained sections of the EHBD, IHBD, and gallbladder from *PK-CK19*^*CreERT*^ mice at 28 days after TAM administration. (B) H&E-stained sections of the EHBD from *PK-CK19*^*CreERT*^ mice at the indicated time points after TAM administration. (C) Venn diagram illustrating the histological phenotypes associated with each oncogenic alteration. (D) H&E-stained sections of the EHBD from *P-CK19*^*CreERT*^ mice at the indicated time points after TAM administration (n = 5–6 mice per time point). (E) ISH for *Tgfb1* in the EHBD of WT and *P-CK19*^*CreERT*^ mice at 30 days after TAM administration. (F) Double staining combining FISH for *Tgfb1* and immunofluorescence for CD45 in the EHBD of *P-CK19*^*CreERT*^ mice, together with IHC staining for F4/80 using serial sections. (G) Comparison of the growth rates of TGFβR2-KO and control organoids in the presence or absence of TGFβ1 (n = 3 per group). Growth was significantly suppressed only in control organoids treated with TGFβ1. Statistical significance was assessed using one-way ANOVA followed by Tukey’s multiple comparisons test. (H) Immunostaining of the indicated proteins in each mouse strain at 15 days after TAM administration (n = 5 per group). Scale bars, 100 μm, except for organoid images (200 μm). BEC, biliary epithelial cell; BTC, biliary tract cancer; eCCA, extrahepatic cholangiocarcinoma; EHBD, extrahepatic bile duct; IHBD, intrahepatic bile duct; ISH, *in situ* hybridization; KO, knockout; TAM, tamoxifen; WT, wild-type.

Next, we performed a time-course analysis of tumor development in *PK-CK19*^*CreERT*^ mice. Papillary outgrowths of surface BECs appeared as early as 10 days after TAM administration and became progressively more prominent by Days 15 and 20, exhibiting an intraductal-growing pattern (Fig. 2B). These findings suggest that surface BECs undergo malignant transformation and directly lead to eCCA in this model. Together, these results indicate that distinct oncogenic alterations affect not only tumor initiation but also the histological phenotype and anatomical distribution of eCCA.

Among the three eCCA mouse models, *PT-CK19*^*CreERT*^ and *KTC-CK19*^*CreERT*^ mice exhibited tumor formation with an infiltrating growth pattern from proliferating PBGs.^13^ In contrast, *PK-CK19*^*CreERT*^ mice developed intraductal-growing tumors originating from luminal epithelial proliferation. These findings suggest that TGFβR2 loss drives the infiltrating growth pattern from PBGs (Fig. 2C). Interestingly, time-course analysis of TGFβR2–wild-type (WT) *P-CK19*^*CreERT*^ mice revealed immune cell infiltration in the stroma surrounding PBGs at Day 30 after TAM administration (Fig. 2D), and *Tgfb1* messenger RNA assessed by ISH was markedly increased in stromal cells around PBGs in *P-CK19*^*CreERT*^ mice compared with Cre-negative WT mice at this time point (Fig. 2E). Double staining by fluorescent ISH for *Tgfb1* and immunofluorescence for CD45 confirmed that TGFβ was predominantly expressed in CD45-positive immune cells (Fig. 2E,F). Serial section staining further revealed abundant infiltration of F4/80-positive macrophages around PBGs in *P-CK19*^*CreERT*^ mice, suggesting that TGFβ secreted from surrounding macrophages may inhibit the abnormal proliferation of PBG cells (Fig. 2F).

To assess the role of TGFβ *in vitro*, we cultured EHBD organoids from *Tgfbr2*^*flox/flox*^ mice and transduced them with either Cre-expressing or control lentivirus to induce *Tgfbr2* deletion (Fig. S1A). No significant difference in growth was observed between control and TGFβR2 KO organoids under basal conditions. However, proliferation of control organoids was markedly suppressed upon TGFβ stimulation, whereas TGFβR2 KO organoids remained unaffected (Fig. 2G). Consistent with these *in vitro* findings, neoplastic expansion of PBGs was suppressed in TGFβR2–WT *P-CK19*^*CreERT*^ mice from Day 30 onward (Fig. 2D). This suggests that TGFβ signaling through TGFβR2 may restrain the proliferation of BECs, particularly that of stem/progenitor cells residing in PBGs.

To further determine why papillary lesions observed in the bile duct lumen regressed in *P-CK19*^*CreERT*^ and *PT-CK19*^*CreERT*^ mice (Fig. 1, Fig. 2D), whereas *PK-CK19*^*CreERT*^ mice developed intraductal-growing CCA (Fig. 2B), we analyzed early lesions 15 days after TAM administration across the three mouse strains. A previous study on prostate cancer demonstrated that PTEN loss alone induces oncogene-induced senescence, thereby restraining tumorigenesis. In contrast, HER2 overexpression suppresses this senescence program and promotes malignant transformation by activating the ERK pathway.^31^ Consistent with these findings, the expression of the senescence marker p21 was markedly increased in the luminal BECs of *P-CK19*^*CreERT*^ and *PT-CK19*^*CreERT*^ mice. In contrast, *PK-CK19*^*CreERT*^ mice showed reduced p21 expression, accompanied by increased phosphorylation of ERK, a major downstream effector of Kras signaling (Fig. 2H). These findings suggest that the additional Kras mutations promote intraductal tumor development by overcoming PTEN loss-induced senescence in luminal BECs through ERK activation.

### Role of the SCAP/SREBP pathway in PTEN loss-driven eCCA development

To further elucidate the mechanisms underlying eCCA development in *PT-CK19*^*CreERT*^ mice, we established EHBD organoids from *Pten*^*flox/flox*^/*Tgfbr2*^*flox/flox*^ mice and generated PTEN/TGFβR2 double-KO (PT) organoids using Cre-expressing lentivirus (Fig. S1B). PT organoids exhibited a significantly higher growth rate than control organoids (Fig. 3A). Gene set enrichment analysis (GSEA) of RNA sequencing (RNA-seq) data using the HALLMARK gene sets revealed significant upregulation of lipid biosynthesis-related pathways, including adipogenesis and fatty acid metabolism, in PT organoids compared with control organoids (Fig. 3B). Consistently, eCCAs in *PT-CK19*^*CreERT*^ mice exhibited an increased expression of sterol regulatory element-binding protein 1 (SREBP1), a master transcriptional regulator of lipogenesis, *in vivo* (Fig. 3C). These results were consistent with the established regulation of SREBP1 by the PI3K pathway.^32^ Increased SREBP1 expression was also observed in eCCA tissues from *PK-CK19*^*CreERT*^ mice (Fig. 3C), indicating that SREBP activation is a common feature of PTEN loss-driven eCCA across distinct oncogenic contexts.

**Fig. 3:**
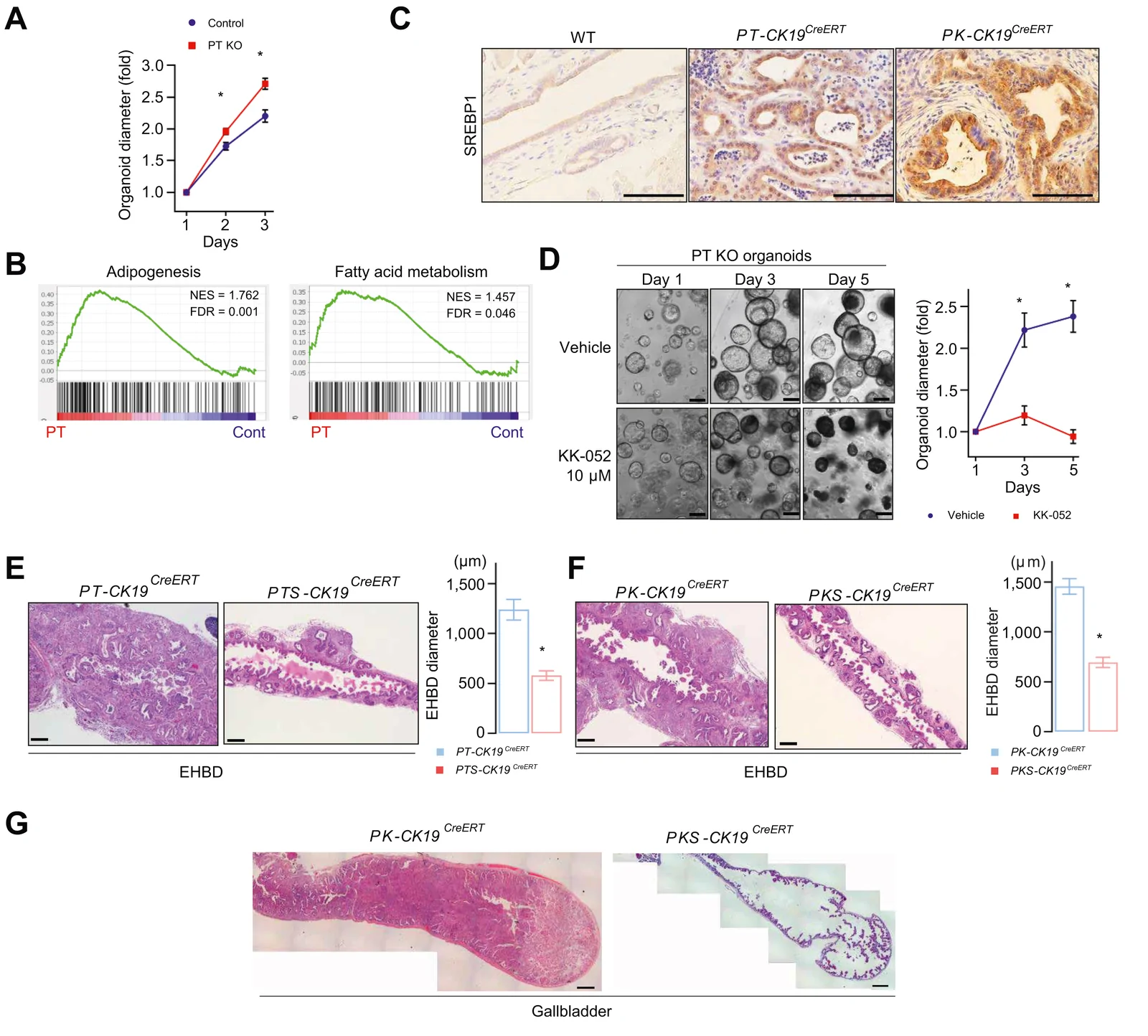
Role of the SCAP/SREBP pathway in PTEN loss-driven eCCA development. (A,B) Comparison of the growth rates (A) and GSEA enrichment (as determined based on RNA-seq data) (B) of PT and control organoids (n = 3 per group). (C) SREBP1 immunostaining in the EHBD from WT, *PT-CK19*^*CreERT*^, and *PK-CK19*^*CreERT*^ mice. (D) Effects of the SREBP inhibitor KK-052 on the growth of PT organoids (n = 3 per group). (E–G) Effects of BEC-specific SCAP deletion on eCCA development in *PT-CK19*^*CreERT*^ (E) and *PK-CK19*^*CreERT*^ mice (F,G) at 90 and 24 days after TAM administration, respectively. (E,F) H&E-stained sections of the EHBD. Bar graphs show the maximum diameter of the EHBD (n = 10 per group). (G) Representative H&E-stained sections of the gallbladder from *PK-CK19*^*CreERT*^ and *PKS-CK19*^*CreERT*^ mice. Scale bars, 100 μm, except for organoid (200 μm) and gallbladder (500 μm) images. All statistical comparisons were performed using Student’s *t*-test. eCCA, extrahepatic cholangiocarcinoma; EHBD, extrahepatic bile duct; FDR, false discovery rate; GSEA, gene set enrichment analysis; NES, normalized enrichment score; RNA-seq, RNA sequencing; SCAP, SREBP cleavage-activating protein; SREBP, sterol regulatory element-binding protein; TAM, tamoxifen; WT, wild-type.

SREBP-mediated lipogenesis promotes carcinogenesis through various mechanisms, such as the provision of membrane lipid components, the activation of oncogenic signaling via lipid mediators, and the reprogramming of cellular energy metabolism.^33^ We previously developed a novel vitamin D-based SREBP inhibitor (KK-052) that induces the degradation of the escort protein SREBP cleavage-activating protein (SCAP).^34^ This protein is essential for SREBP activation. Therefore, its degradation suppressed SREBP activity. Treatment with KK-052 markedly suppressed the growth of the PT organoids, suggesting the involvement of the SCAP/SREBP pathway in eCCA development (Fig. 3D). To further validate the role of this pathway *in vivo*, we crossed *PT-CK19*^*CreERT*^ mice with *Scap*^*flox/flox*^ mice (*PTS-CK19*^*CreERT*^). Although BEC-specific SCAP deletion alone did not induce apparent histological changes in the EHBD for up to 90 days after TAM administration (data not shown), eCCA development was almost completely abrogated in *PTS-CK19*^*CreERT*^ mice (Fig. 3E). To determine whether this requirement for SCAP/SREBP signaling was conserved across distinct PTEN loss-driven eCCA models, we also examined *PK-CK19*^*CreERT*^ mice. Consistently, both eCCA and GBC formation were significantly suppressed when *PK-CK19*^*CreERT*^ mice were crossed with *Scap*^*flox/flox*^ mice (*PKS-CK19*^*CreERT*^) (Fig. 3F,G). Taken together, these results indicate that the SCAP/SREBP pathway is a critical driver of PTEN loss-driven eCCA development.

### Differential role of the SCAP/SREBP pathway in iCCA development

We examined the effects of SCAP deletion on iCCA development. Although SCAP deficiency in *PK-CK19*^*CreERT*^ mice attenuated iCCA development in large IHBDs, peripheral iCCA lesions appeared to be exacerbated (Fig. 4A). Consistently, CK19 immunostaining showed a relative decrease in CK19-positive areas in large IHBDs and an increase in peripheral IHBDs in *PKS-CK19*^*CreERT*^ mice compared with those in *PK-CK19*^*CreERT*^ mice (Fig. 4B). Because other factors, such as EHBD obstruction, may affect iCCA development in this model, we examined the role of the SCAP/SREBP pathway in a liver-specific genetically engineered mouse model. To this end, we generated a previously described iCCA model by crossing *Pten*^*flox/flox*^/*Kras*^*G12D*^ mice with *Albumin-Cre* mice (*PK-Alb*)^8^ and compared them with mice lacking SCAP (*Pten*^*flox/flox*^/*Kras*^*G12D*^*/Scap*^*flox/flox*^*/Albumin-Cre* mice; *PKS-Alb*). All *PKS-Alb* mice developed severe jaundice and died by 5 weeks of age, whereas all *PK-Alb* mice survived at this time point *(PKS-Alb*: n = 8; *PK-Alb*: n = 10, *p* <0.0001). Histological analysis at 4 weeks of age revealed that, although *PK-Alb* mice had only begun to develop iCCA lesions in large IHBDs, *PKS-Alb* mice exhibited highly aggressive iCCA that diffusely invaded the entire liver (Fig. 4C). Quantitative analysis of CK19 immunostaining revealed a significantly increased CK19-positive area in *PKS-Alb* mice compared with that in *PK-Alb* mice (Fig. 4D). These findings suggest that the biological function of the SCAP/SREBP pathway differs according to the anatomical origin of the BTC.

**Fig. 4:**
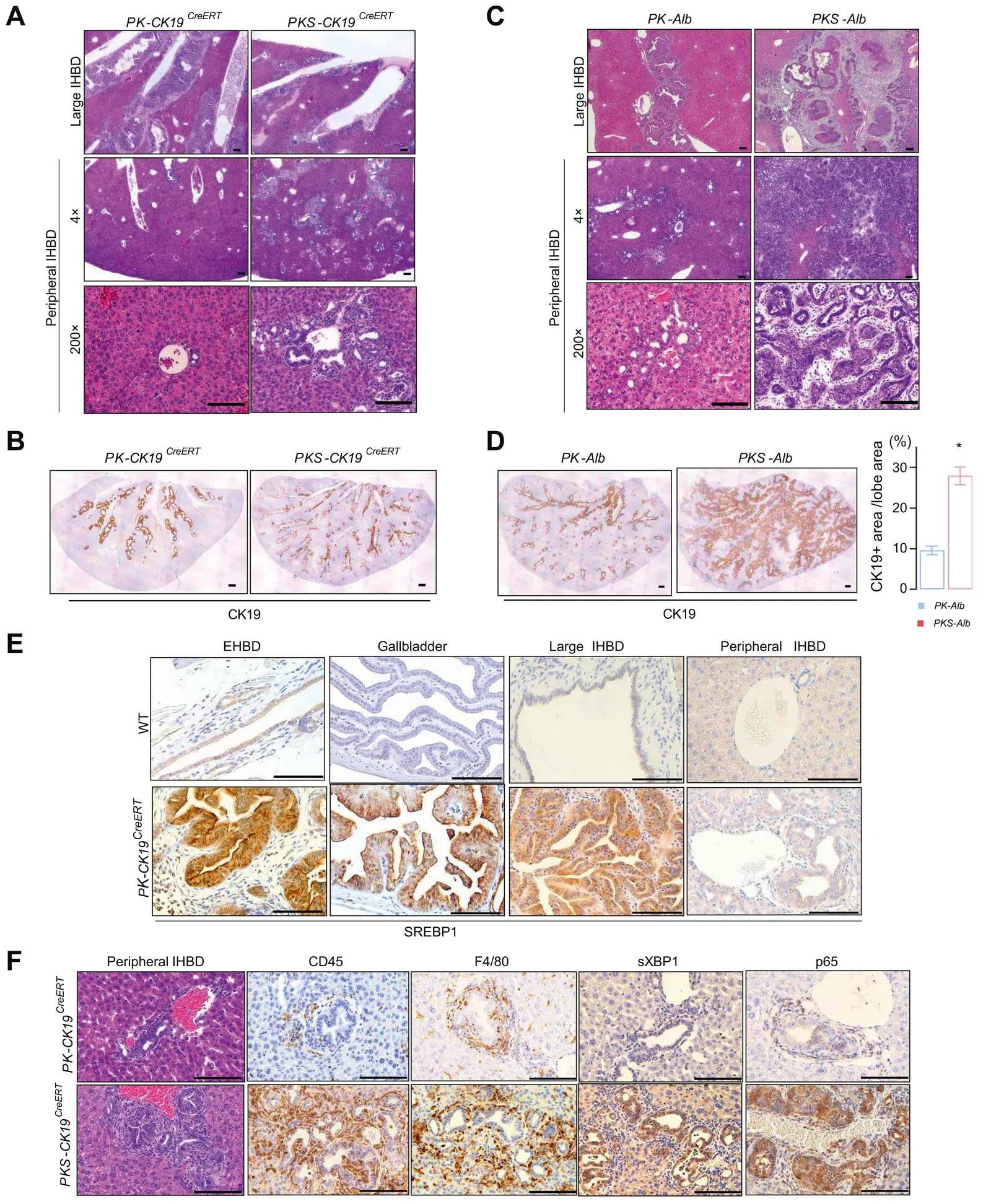
Effect of SCAP deletion on iCCA development. (A) Representative H&E-stained sections of large and peripheral IHBDs from *PK-CK19*^*CreERT*^ and *PKS-CK19*^*CreERT*^ mice at 24 days after TAM administration. (B) Tile scan images of CK19 IHC in the livers of mice shown in (A). (C) Representative H&E-stained sections of large and peripheral IHBDs from *PK-Alb* and *PKS-Alb* mice at 4 weeks of age. (D) Tile scan images of CK19 IHC in the livers of mice shown in (C). The bar graph shows the CK19-positive area (n = 5 per group). Statistical significance was assessed using Student’s *t*-test. (E) SREBP1 immunostaining in different regions of the biliary tree in WT and *PK-CK19*^*CreERT*^ mice at 4 weeks after TAM administration. (F) H&E staining and IHC for the indicated proteins in peripheral IHBDs from *PK-CK19*^*CreERT*^ and *PKS-CK19*^*CreERT*^ mice at 4 weeks after TAM administration. Scale bars, 100 μm, except for tile scan images (500 μm). iCCA, intrahepatic cholangiocarcinoma; IHBD, intrahepatic bile duct; IHC, immunohistochemistry; SCAP, SREBP cleavage-activating protein**;** SREBP, sterol regulatory element-binding protein**;** TAM, tamoxifen; WT, wild-type.

To investigate the mechanisms underlying the distinct effects of SCAP deficiency according to the anatomical location of BTC, we first examined SREBP1 activation in each CCA subtype in *PK-CK19*^*CreERT*^ mice. IHC analysis revealed marked upregulation and nuclear localization of SREBP1 in eCCA, GBC, and large-duct iCCA, whereas no SREBP1 activation was detected in peripheral iCCA (Fig. 4E). Thus, the limited dependence of peripheral iCCA on SREBP1 signaling may explain the reduced tumor-suppressive effect of SCAP deletion.

We further investigated the mechanism underlying the paradoxical acceleration of peripheral iCCA following SCAP deletion. Peripheral iCCA lesions in *PKS-CK19*^*CreERT*^ mice exhibited prominent inflammatory cell infiltration compared with those in *PK-CK19*^*CreERT*^ mice, with abundant CD45-positive leukocytes and F4/80-positive macrophages (Fig. 4F). SCAP deficiency alters the endoplasmic reticulum (ER) membrane composition and induces ER stress in the liver.^22^ Consistent with this finding, the expression of sXBP1, a marker of ER stress, was markedly increased in the peripheral iCCA lesions of *PKS-CK19*^*CreERT*^ mice. In addition, expression and nuclear accumulation of NF-κB subunit p65, which can be promoted by XBP1 signaling,^35^^,^^36^ were increased in these lesions (Fig. 4F). NF-κB activation in cancer cells promotes macrophage recruitment through chemokine induction and facilitates tumor progression.^37^ Collectively, SCAP deficiency is associated with activation of the ER stress/XBP1/NF-κB pathway and increased macrophage infiltration, which may contribute to the aggressive phenotype observed in this model.

### SCAP/SREBP pathway activation links lipid metabolism to ferroptosis in eCCA

Although the SCAP/SREBP pathway represents a promising therapeutic target for eCCA, it cannot be readily exploited when the distinction between iCCA and eCCA is ambiguous. Moreover, the SCAP/SREBP pathway is essential for maintaining organ homeostasis, and its long-term suppression may lead to unexpected adverse effects.^22^ To identify alternative therapeutic targets, we established EHBD organoids from *Pten*^*flox/flox*^/*Tgfbr2*^*flox/flox*^*/Scap*^*flox/flox*^ mice and generated PTEN/TGFβR2/SCAP triple-KO (PTS) organoids using Cre-expressing lentivirus (Fig. S1C). The PTS organoids showed significantly slower growth than the PT organoids (Fig. 5A). We then performed RNA-seq analysis and compared the gene expression profiles of the control, PT, and PTS organoids. Ferroptosis-related gene sets derived from WikiPathways were upregulated in the PT organoids, whereas this induction was markedly suppressed in the PTS organoids (Fig. 5B).

**Fig. 5:**
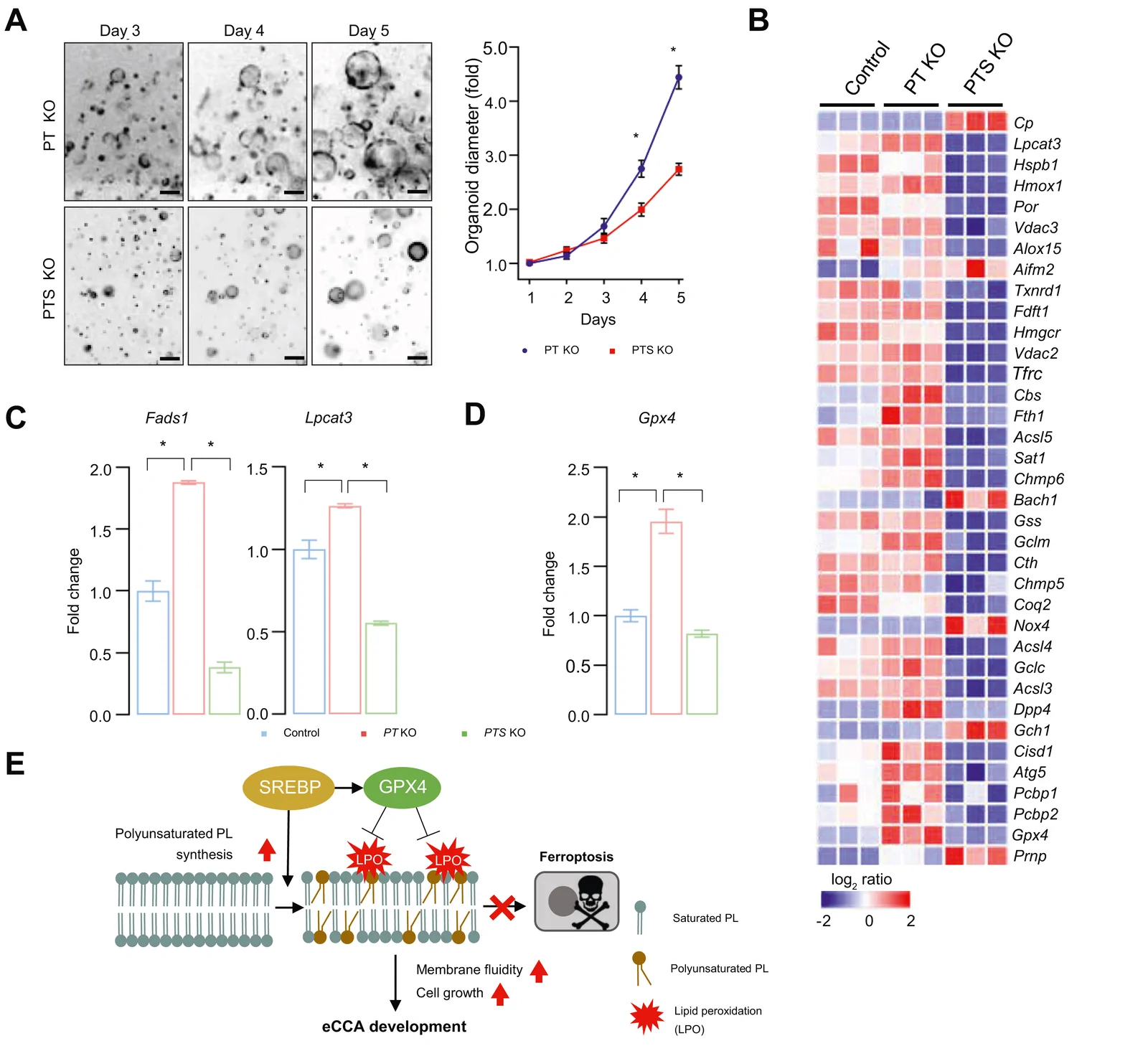
SCAP/SREBP pathway activation links lipid metabolism to ferroptosis in eCCA. (A) Comparison of the growth rates of PT and PTS organoids (n = 3 per group) (scale bar, 200 μm). Statistical significance was assessed using Student’s *t*-test. (B) Heatmap showing the expression of ferroptosis-related genes in control, PT, and PTS organoids, as determined by RNA-seq and derived from WikiPathways. (C,D) Expression levels of the indicated genes in control, PT, and PTS organoids (n = 3 per group). Statistical significance was assessed using one-way ANOVA followed by Tukey’s multiple comparisons test. (E) Schematic model of SREBP-mediated eCCA development. SREBP-activated tumor cells promote tumor progression through PUFA-enriched PL synthesis while simultaneously evading PUFA-mediated ferroptosis via the upregulation of GPX4 expression. eCCA, extrahepatic cholangiocarcinoma; GPX4, glutathione peroxidase 4; KO, knockout; PL, phospholipid; PUFA, polyunsaturated fatty acid; RNA-seq, RNA sequencing; SCAP, SREBP cleavage-activating protein; SREBP, sterol regulatory element-binding protein.

Ferroptosis is a form of programmed cell death driven by the lipid peroxidation of membrane phospholipids and is catalyzed by intracellular free iron. Phospholipids containing polyunsaturated fatty acids (PUFAs) are particularly susceptible to peroxidation,^38^ and SREBP activation enhances the generation of PUFA-containing phospholipids.^22^ Consistent with this, PT organoids showed increased expression of *Fads1*, which is involved in PUFA synthesis, and *Lpcat3*, which incorporates PUFAs into phospholipids. However, the expression of these enzymes was significantly downregulated in PTS organoids (Fig. 5C). PUFA-containing phospholipids enhance membrane fluidity and cellular stress tolerance, thereby conferring a growth advantage to cancer cells;^39^ however, their accumulation also increases susceptibility to ferroptosis.

Cells also possess intrinsic defense mechanisms against ferroptosis, the most important of which is the glutathione peroxidase 4 (GPX4)–glutathione (GSH) system. GPX4 reduces lipid peroxides in a GSH-dependent manner and prevents ferroptosis.^38^ Notably, *Gpx4* expression was significantly upregulated in PT organoids but downregulated in PTS organoids (Fig. 5D). This finding is consistent with recent studies demonstrating that GPX4 is a direct transcriptional target of SREBP.^40^^,^^41^ These results suggest that SREBP-activated eCCA cells evade ferroptosis induced by enhanced PUFA-enriched phospholipid synthesis through upregulation of GPX4 expression (Fig. 5E). Therefore, targeting GPX4 may be a promising therapeutic strategy for the treatment of PTEN loss-driven eCCA.

### Therapeutic potential of GPX4 inhibition

To evaluate the therapeutic potential of GPX4 inhibition, we used the *PK-CK19*^*CreERT*^ mouse model, which rapidly develops eCCA. EHBD organoids were established from *Pten*^*flox/flox*^/*Kras*^*G12D*^ mice and were subjected to Cre-mediated recombination (PK organoids) (Fig. S1D). Similar to the PT organoids, the PK organoids exhibited increased expression of *Fads1*, *Lpcat3*, and *Gpx4* (Fig. 6A). Treatment with the GPX4 inhibitor RSL3 significantly reduced the viability of PK organoids and enhanced lipid peroxidation, as detected using a fluorescent probe (Fig. 6B,C). Furthermore, *in vivo* administration of RSL3 to *PK-CK19*^*CreERT*^ mice significantly suppressed the development of eCCA and GBC (Fig. 6D) without causing significant changes in body weight or overt deterioration in general condition. Immunostaining for 4-hydroxynonenal protein adducts, a marker of lipid peroxidation, revealed markedly increased levels in eCCA tissues from RSL3-treated *PK-CK19*^*CreERT*^ mice (Fig. 6E). Importantly, RSL3 treatment suppressed iCCA development in large IHBDs without exacerbating peripheral iCCA lesions, indicating a favorable therapeutic window (Fig. 6D).

**Fig. 6:**
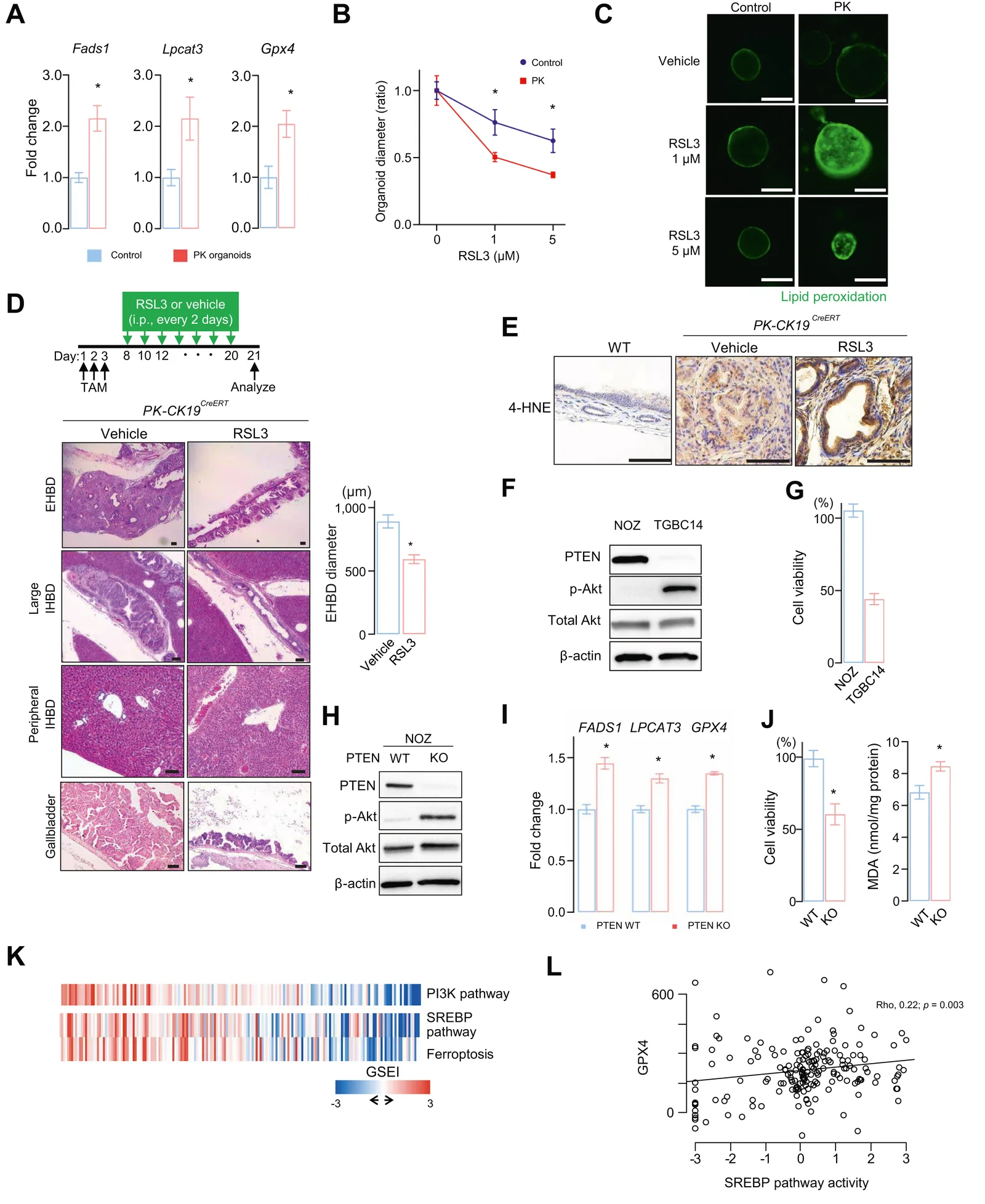
Therapeutic potential of GPX4 inhibition. (A) Expression levels of the indicated genes in control and PK organoids, as determined by real-time PCR (n = 3 per group). (B,C) Effects of the GPX4 inhibitor RSL3 on PK and control organoids. The graph shows the organoid diameter at 2 days after RSL3 treatment, expressed as the ratio relative to the pretreatment baseline (n = 3 per group) (B), and lipid peroxidation was detected using a fluorescent probe (C). (D) Effects of RSL3 on eCCA, iCCA, and GBC development in *PK-CK19*^*CreERT*^ mice. Representative H&E-stained sections are shown. The bar graph shows the maximum diameter of the EHBD (n = 5 per group). (E) IHC staining for 4-HNE in the EHBD from WT and *PK-CK19*^*CreERT*^ mice treated with RSL3 or the vehicle. (F) Western blot analyses of indicated proteins in NOZ and TGBC14 cells. (G) Viability of NOZ and TGBC14 cells treated with RSL3 for 24 h (n = 6 per group). (H–J) Experiments using PTEN-KO NOZ cells generated by the CRISPR–Cas9 system. (H) Western blot analyses confirming PTEN-KO and increased Akt phosphorylation. (I) Expression levels of the indicated genes in WT and PTEN-KO NOZ cells, as determined by real-time PCR (n = 3 per group). (J) Viability of and lipid peroxidation in WT and PTEN-KO NOZ cells treated with RSL3 for 24 h (n = 3 per group). All statistical comparisons were performed using Student’s *t*-test. (K) Heatmap showing associations among PI3K, SREBP, and ferroptosis pathway activities, based on RNA-seq data from 182 human eCCA samples. (L) Scatter plot showing the relationship between GPX4 expression and SREBP pathway activity in human eCCA samples. Scale bar, 100 μm. 4-HNE, 4-hydroxynonenal; eCCA, extrahepatic cholangiocarcinoma; EHBD, extrahepatic bile duct; GBC, gallbladder cancer; GPX4, glutathione peroxidase 4; iCCA, intrahepatic cholangiocarcinoma; IHC, immunohistochemistry; KO, knockout; RNA sequencing**;** SREBP, sterol regulatory element-binding protein; WT, wild-type.

To further explore the potential clinical applications of our findings, we examined the response to GPX4 inhibition in human BTC cell lines. We first compared the sensitivity to RSL3 between NOZ cells, a GBC cell line known to retain PTEN expression, and another GBC cell line, TGBC14, which harbors PTEN loss^42^ (Fig. 6F). As expected, TGBC14 cells exhibited a significantly greater reduction in viability after RSL3 treatment than the NOZ cells (Fig. 6G). Furthermore, we generated PTEN-KO NOZ cells using the CRISPR–Cas9 system to directly assess whether PTEN loss enhanced sensitivity to RSL3 (Fig. 6H). Increased Akt phosphorylation and upregulation of *LPCAT3*, *FADS1*, and *GPX4* expression were observed in these PTEN-KO NOZ cells (Fig. 6H,I). PTEN-KO NOZ cells displayed significantly increased sensitivity to RSL3, accompanied by enhanced malondialdehyde (MDA) accumulation, an end-product of lipid peroxidation (Fig. 6J). Consistent with these findings, PTEN knockdown in TFK-1 cells, a PTEN-expressing eCCA cell line, also increased Akt phosphorylation, upregulated ferroptosis-related genes, and enhanced sensitivity to RSL3 (Fig. S2A–C). These results support the hypothesis that PTEN-deficient BTCs are particularly vulnerable to ferroptosis-inducing agents.

Finally, we analyzed public expression data from human eCCA samples (n = 182) to examine the clinical relevance of our findings.^28^ PI3K, SREBP, and ferroptosis pathway signatures were highly correlated (Fig. 6K). In addition, GPX4 expression was significantly associated with activation of the SREBP pathway (Fig. 6L). These findings support the relevance of our experimental observations in human eCCA.

## Discussion

In this study, we investigated the genetic and metabolic determinants of BTC development using a series of genetically engineered mouse models. Our findings revealed that the combination and cellular context of oncogenic alterations in BECs critically dictate tumor initiation, growth patterns, and anatomical distribution. We identified SCAP/SREBP-driven lipid metabolic reprogramming as a key determinant of PTEN loss-driven eCCA progression. Furthermore, this metabolic dependency confers ferroptosis-associated vulnerability, providing a mechanistic rationale for the targeting of GPX4 as a potential therapeutic strategy.

Our findings demonstrate that the loss of TGFβR2 plays a more prominent role in promoting eCCA than in iCCA development. This observation is consistent with our previous report showing that BEC-specific Kras activation combined with deletion of TGFβR2 and E-cadherin drives eCCA development.^13^ The present study further suggests that TGFβ signaling is particularly important for restraining the proliferation of PBG cells and that disruption of this pathway promotes the development of periductal infiltrating-type eCCA originating from PBGs. Although it remains unclear whether a similar histological phenotype is observed in human eCCA, the mutation frequency of TGFβ receptors in human BTC is relatively low (∼3–5%),^5^^,^^6^ highlighting the need for validation in large-scale clinical datasets. A previous study using the same *PT-CK19*^*CreERT*^ mice demonstrated that cholangiocyte injury via a 3,5-diethoxycarbonyl-1,4-dihydro-collidine diet promoted iCCA development,^43^ suggesting that additional inflammatory or injurious insults may increase the susceptibility of PTEN/TGFβR2-deficient IHBDs to iCCA formation. In addition, we cannot exclude the possibility that the recombination efficiency of *Tgfbr2* is lower in IHBDs, which may have partially contributed to the absence of iCCA development in *PT-CK19*^*CreERT*^ mice.

Another intriguing finding was the distinct fate of the early papillary lesions in the bile duct lumen. Although luminal papillary lesions induced by PTEN loss regressed over time, additional Kras activation facilitated the development of an intraductal-growing CCA. Our findings suggest that PTEN loss induces cellular senescence in luminal BECs, whereas Kras activation may overcome this barrier through ERK signaling, consistent with previous findings in prostate cancer.^31^ Although further mechanistic studies are required, these findings provide a potential explanation for the divergent histological evolution of luminal biliary lesions following PTEN loss alone *vs*. combined PTEN loss and Kras activation.

Although PTEN loss alone did not induce eCCA in our adult-onset *P-CK19*^*CreERT*^ mouse model, a recent report showed that embryonic deletion of PTEN using *Pdx1-Cre* was sufficient to drive eCCA development.^44^ This discrepancy may be attributable to the timing of PTEN loss during biliary development. Embryonic PTEN deletion may alter biliary differentiation programs or expand susceptible progenitor populations, whereas adult-onset PTEN loss in differentiated BECs and PBGs likely requires additional oncogenic or inflammatory stimuli to initiate malignant transformation.

Recent studies have increasingly highlighted metabolic reprogramming as a hallmark of cancer; alterations in lipid metabolism support tumor growth and survival.^45^ In the present study, lipogenesis was markedly activated in PTEN-deficient eCCA in association with SREBP activation. Moreover, the inhibition of SCAP, a critical regulator of SREBP activation, substantially suppressed the development of both eCCA and GBC. These findings indicated that SREBP-mediated lipogenesis is a key driver of extrahepatic BTC development. In contrast, genetic ablation of SCAP was associated with exacerbation of peripheral iCCA lesions. This finding was further corroborated using a liver-specific genetically engineered mouse model. This observation suggests that the role of SCAP in BTC pathogenesis differs depending on the anatomical subtype. Consistent with this hypothesis, peripheral iCCA exhibited no SREBP1 activation in the present study despite PTEN loss, suggesting a reduced dependence on SREBP-mediated lipogenesis compared to eCCA, GBC, and large-duct iCCA. In addition, SCAP deficiency in peripheral iCCA was accompanied by activation of the XBP1/NF-κB pathway and increased macrophage infiltration, which may contribute to the aggressive phenotype observed in this model. However, the precise molecular mechanisms remain to be fully elucidated and warrant further investigation.

The SCAP/SREBP pathway represents a potential therapeutic target in eCCA. However, complete or long-term inhibition of this pathway may not be feasible in clinical settings owing to its essential role, as shown in our previous study on metabolic dysfunction-associated steatotic liver disease (MASLD).^22^ Moreover, the effect of SCAP deletion differs between extrahepatic and intrahepatic BTC, further suggesting that systemic blockade of this pathway may have unintended context-dependent consequences. Therefore, we explored the downstream vulnerabilities associated with SREBP activation in PTEN-deficient eCCA. Transcriptomic profiling revealed selective upregulation of the expression of ferroptosis-related genes in PT organoids, suggesting that PTEN/SREBP-driven eCCA relies on ferroptosis resistance mechanisms for survival. Therefore, we focused on GPX4, a key regulator of ferroptosis, as a potential therapeutic target. SREBP-driven lipogenesis increases PUFA-containing phospholipids, which are highly susceptible to lipid peroxidation, thereby requiring robust ferroptosis defense. Thus, SREBP activation represents a ‘double-edged sword’ for tumor cells, simultaneously promoting tumor growth while creating a dependency on ferroptosis resistance mechanisms. Consistent with previous reports identifying GPX4 as a direct transcriptional target of SREBP,^40^^,^^41^ GPX4 expression was markedly downregulated upon SCAP deletion in this study. Importantly, the pharmacological inhibition of GPX4 using RSL3 significantly suppressed eCCA, GBC, and large-duct iCCA development without exacerbating peripheral iCCA lesions *in vivo*. Because suitable paired eCCA cell lines with comparable PTEN status were not available, we initially used GBC cell lines for mechanistic analyses and subsequently validated the key findings in TFK-1 human eCCA cells following PTEN knockdown. However, further validation in additional human eCCA cell lines is required to establish the generalizability of these findings. Taken together, these findings identified a metabolic vulnerability in which PTEN/SREBP-driven extrahepatic cholangiocarcinogenesis depends on GPX4-mediated ferroptosis resistance for tumor survival, supporting the applicability of GPX4 inhibition as a potential therapeutic strategy. In addition to its direct tumor-suppressive effects, ferroptosis has been implicated in the remodeling of the tumor microenvironment through immunogenic cell death and the modulation of antitumor immunity.^46^^,^^47^ Therefore, the potential synergy between ferroptosis induction and immune checkpoint blockade in eCCA requires further investigation.

Analysis of human eCCA clinical samples further revealed that activation of the PI3K–SREBP axis is strongly correlated with ferroptosis-related gene signatures and that SREBP expression is positively correlated with GPX4 levels. PTEN loss and PI3K pathway activation are observed in ∼10% of human eCCA cases, and Akt/mTOR pathway activation is particularly enriched in distal eCCA.^5^^,^^6^^,^^28^ Thus, the PTEN/PI3K/SREBP–GPX4 axis may represent a candidate target in biomarker-defined subsets of human BTC. However, the systemic inhibition of GPX4 remains a major challenge in clinical applications. GPX4 is indispensable for the survival and function of multiple normal cell types, including neurons, renal cells, and immune cells, suggesting that systemic GPX4 inhibition may result in significant on-target toxicity.^48^^,^^49^ Therefore, successful clinical translation will likely require strategies that improve tumor specificity, such as tumor-selective drug delivery, to minimize toxicity in healthy tissues. In addition, other components of the ferroptosis regulatory network should be considered alternative or complementary therapeutic targets.

In conclusion, distinct oncogenic mutations shape the histological and anatomical diversity of BTC, and the SCAP/SREBP pathway plays a critical role in PTEN loss-driven extrahepatic cholangiocarcinogenesis. Targeting GPX4-dependent ferroptosis resistance is a promising therapeutic strategy for these tumors.

## Financial support

HN is supported by grants from JSPS KAKENHI Grant Number 24K02436; AMED Grant Numbers JP25fk0210130, JP26fk0210195, JP26fk0210149, JP26fk0210178, and JP266f0137007; the Princess Takamatsu Cancer Research Fund; the Daiichi Sankyo Foundation of Life Science; and the Takeda Science Foundation. HT is supported by JSPS KAKENHI Grant Number 26K10876. TY is supported by JSPS KAKENHI Grant Number 20K08376. YH is supported by JSPS KAKENHI Grant Number 25K11250 and the Ekiden for Life Cancer Research Encouragement Award.

## Authors’ contributions

Performed the experiments, analyzed the data, and wrote the paper YH. Helped with some experiments and interpretation of the data and edited the manuscript: YM, MT, SN, TY, SK, KI, GK, YT, HT, AE, HG. Analyzed human data: NF. Conceived and designed the study and wrote the paper: HN.

## Abbreviations

4-HNE, 4-hydroxynonenal; BEC, biliary epithelial cell; BTC, biliary tract cancer; CCA, cholangiocarcinoma; eCCA, extrahepatic cholangiocarcinoma; EHBD, extrahepatic bile duct; ER, endoplasmic reticulum; GBC, gallbladder cancer; GPX4, glutathione peroxidase 4; GSEA, gene set enrichment analysis; GSH, glutathione; iCCA, intrahepatic cholangiocarcinoma**;** IHBD, intrahepatic bile duct; IHC, immunohistochemistry; ISH, *in situ* hybridization; KO, knockout; MASLD, metabolic dysfunction-associated steatotic liver disease; MDA, malondialdehyde; PBG, peribiliary gland; PUFA, polyunsaturated fatty acid; RNA-seq, RNA sequencing; SCAP, SREBP cleavage-activating protein; SREBP, sterol regulatory element-binding protein; TAM, tamoxifen; WT, wild-type.

## Conflicts of interest

The authors declare no conflicts of interest.

Please refer to the accompanying ICMJE disclosure forms for further details.
