# Supplementary material for: SREBP-driven lipogenesis coupled with ferroptosis resistance as a therapeutic target in PTEN-loss extrahepatic cholangiocarcinoma
Source: JHEP Rep. 2026 Aug 29;8(11):102015. doi: 10.1016/j.jhepr.2026.102015 (PMC13693315; doi:10.1016/j.jhepr.2026.102015)
Supplement: Supplementary file 1 [file mmc1.pdf]

SREBP-driven lipogenesis coupled with ferroptosis resistance as a therapeutic target in PTEN-loss extrahepatic cholangiocarcinoma

Yuki Hayata, Yuki Matsushita, Mina Tempaku, Shun Nakagawa, Tomoharu Yamada, Satoshi Kawamura, Kiyora Izuoka, Genki Kimura, Yutaro Tachi, Hideaki Tanaka, Akiko Eguchi, Naoto Fujiwara, Hidemasa Goto, Hayato Nakagawa

Table of contents

Supplementary Materials and Methods…………………………………………………..2

Supplementary References……………………………………………………………..5

Table S1…………………………………………………………………………………...6

Fig. S1……………………………………………………………………………………..7

Fig. S2……………………………………………………………………………………..8

Supplementary Materials and Methods

Animal experiments

Tamoxifen (TAM) (Sigma) was dissolved in corn oil containing 10 % ethanol and administered by oral gavage to mice 7–8 weeks of age. RSL3 was dissolved in DMSO at 50 mg/mL, diluted to 5 mg/mL in corn oil, and administered intraperitoneally to mice at a dose of 50 mg/kg every other day. Both male and female mice were used during the initial characterization of the mouse models. Although no obvious sex differences in tumor development were observed, male mice were subsequently used for time-course and comparative experiments to minimize experimental variability.

Histological analyses

Liver and bile duct tissues were processed for H&E staining and immunostaining as described previously (1, 2). For Immunohistochemistry (IHC), fixed and paraffin-embedded liver sections were deparaffinized and incubated in Target Retrieval Solution (Dako) buffer at 95°C for 35 min for antigen retrieval, and then incubated overnight at 4°C with the primary antibodies. Biotinylated secondary antibodies (Pharmingen) were added and incubated for 20 min at room temperature. Streptavidin-horseradish peroxidase (Pharmingen) was added and after 30 min, the sections were developed with 3,3'-diaminobenzidine (DAB) substrate (Vector Laboratories) and counterstained with hematoxylin. The CK19-positive area was quantified using QuPath. For immunofluorescence, slides were incubated with primary antibodies, followed by secondary antibodies labeled with Alexa 488 or 555 (Invitrogen).

For LacZ staining, isolated periampullary tissues were incubated for 2 h in fixative solution [1 % formaldehyde, 0.2 % glutaraldehyde, and 0.02 % NP40 in phosphate-buffered saline (PBS)] at 4 °C. The tissues were washed twice in PBS at room temperature and then incubated in b-galactosidase substrate (5 mM $K_3FE(CN)_6$, 5 mM $K_4Fe(CN)_6.3H_2O$, 2 mM $MgCl_2$, 0.02 % NP-40, 0.1 % Na deoxycholate, and 1 mg/mL X-gal in PBS) in the dark overnight at room temperature. After fixation, sections were counterstained with nuclear fast red.

Biochemical analyses and reagents

The following antibodies were used: anti-PTEN, anti-Akt, anti–phospho-Akt, anti-spliced-XBP1 (sXBP1), anti-phospho-ERK, and anti-p65 (Cell Signaling Technology); anti-CK19 (for immunohistochemistry) and anti-SREBP1 (Merck); anti-Ki67 (GeneTex); anti-F4/80 (Bio-Rad); anti–α-smooth muscle actin, anti-CK-19 (for immunofluorescence), and anti-p21

(Abcam); anti-CD45 (eBioscience); anti-4-HNE (JaICA); and anti–β-actin (BioVision). The relative amount of each mRNA was quantified via real-time PCR and normalized to B2M mRNA expression.

Cell lines

NOZ cells (female donor) were obtained from the JCRB Cell Bank, and TGBC14 (female donor) and TFK-1 (male donor) cells were obtained from RIKEN. The cell lines were cultured in William's E medium (NOZ), DMEM (TGBC14), or RPMI-1640 (TFK-1), each supplemented with 10% fetal bovine serum (FBS). Cell viability was assessed using a Cell Counting Kit-8 (Dojindo). Malondialdehyde (MDA) levels were quantified using a TBARS Assay Kit (Cayman Chemical) according to the manufacturer's instructions. PTEN-specific and control small interfering RNAs (siRNAs) were purchased from Santa Cruz Biotechnology and transfected using RNAiMAX (Invitrogen).

Organoid experiments

Isolated EHBD tissue was cut into small pieces and incubated in dissociation buffer [RPMI with 4 % fetal calf serum, 1 mg/mL collagenase D (Roche), 0.5 mg/mL dispase (Gibco), and 40 μg/mL DNase (Roche)] at 37 °C for 30 min. Incubated EHBD was mixed with Matrigel and seeded onto plates overlaid with culture medium [Ad-DMEM/F12 (Invitrogen) supplemented with 1 mM N-acetylcysteine (Wako), 50 ng/mL EGF, 1 μg/mL Rspo1, 100 ng/mL Noggin, 100 ng/mL FGF10 (Peprotech), and 10 % Wnt3a-conditioned medium (ATCC)]. Passaging was performed at 1:4–1:8 split ratios once per week. For passaging, organoids were incubated on ice in Cell Recovery Solution (BD) for 30 min, passed twice through 29-guage needle, and re-embedded in Matrigel. The ROCK inhibitor Y-27632 (10 μM; Wako) was added to the initial culture and after organoid passaging. All organoids used in this study were established from male mice. To induce gene recombination, organoids were infected with Cre-expressing or control lentivirus, and transduced cells were selected using puromycin. Two weeks later, organoids were used for experiments. Lipid peroxidation in organoids was assessed using the Lipid Peroxidation Probe BODIPY 581/591 C11 (L267; Dojindo).

Generation of PTEN-knockout cells

Gene targeting using the CRISPR–Cas9 system was performed in NOZ cells as described previously (3, 4). Briefly, we used pSpCas9(BB)-2A-Puro V2.0 (pX459, Addgene plasmid ID #62988) carrying a human PTEN-specific guide RNA (gRNA) sequence, 5′-

TTGATGATGGCTGTCATGTC(TGG)-3′, in which the PAM sequence is indicated in parentheses. The 5′ and 3′ homology arms of the human PTEN locus were amplified and cloned into the pMK293 vector (Addgene plasmid ID #72831), in which the neomycin resistance gene had been replaced with a blasticidin resistance gene and the mAID-mCherry2 cassette had been removed to facilitate efficient gene knockout. NOZ cells were co-transfected with the PTEN-targeting pX459 plasmid and the PTEN-knockout donor plasmid at a 1:1 ratio. Following selection with 10 μg/mL blasticidin (FUJIFILM Wako Pure Chemical), antibiotic-resistant clones were isolated.

RNA-seq analyses

Total RNA was extracted from EHBD organoids using ISOGEN with Spin Column (Nippon Gene). Sequencing libraries were prepared using the SMART-Seq™ v4 Ultra™ Low Input RNA Kit (Takara) and the TruSeq RNA Sample Prep Kit v2 (Illumina), and were sequenced on a Novaseq 6000 system (Illumina) according to the manufacturers' protocols. Raw sequencing reads were aligned to the mouse reference genome (mm39) using STAR (PMID: 23104886), and gene expression levels were normalized using the DESeq2 (PMID: 25516281). Pathway enrichment in EHBD organoids was analyzed by gene set enrichment analysis (GSEA) using gene sets from HALLMARK database and WikiPathways (5, 6).

Analyses of human samples

Transcriptome profiles of 182 human extrahepatic cholangiocarcinoma tissues were extracted from NCBI Gene Expression Omnibus database (www.ncbi.nlm.nih.gov/geo, accession number GSE132305) (7). Transcriptional target and pathway member genes of KRAS pathway activity (HALLMARK_KRAS_SIGNALING_UP), TGFb pathway activity (HALLMARK_TGF_BETA_SIGNALING), PI3K pathway activity (HALLMARK_PI3K_AKT_MTOR_SIGNALING), SREBP pathway genes (WP_STEROL_REGULATORY_ELEMENT_BINDING_PROTEINS_SREBP_SIGNALING), and Ferroptosis (WP_FERROPTOSIS) were obtained from Molecular Signature Database (www.broadinstitute.org/msigdb). Modulation of signatures of interest in individual samples was determined using the eseach algorithm (8). Briefly, the eseach algorithm assessed activation status of the target signatures by GSEA, and scored as gene set enrichment index (GSEI) calculated from normalized enrichment score (NES) and gene set enrichment p value based on 1,000-time iterative random gene permutations as follows: GSEI = - sgn(NES) x log10 p value. GSEI of +3 indicates induction at enrichment p = 0.001, GSEI of -3 indicates

suppression (green) at enrichment p = 0.001, and GSEI of 0 indicates no modulation at enrichment p = 1.0.

Supplementary References

1. Nakagawa H, Suzuki N, Hirata Y, Hikiba Y, Hayakawa Y, Kinoshita H, Ihara S, et al. Biliary epithelial injury-induced regenerative response by IL-33 promotes cholangiocarcinogenesis from peribiliary glands. Proc Natl Acad Sci U S A 2017;114:E3806-e3815.
2. Kurosaki S, Nakagawa H, Hayata Y, Kawamura S, Matsushita Y, Yamada T, Uchino K, et al. Cell fate analysis of zone 3 hepatocytes in liver injury and tumorigenesis. JHEP Rep 2021;3:100315.
3. Goto H, Natsume T, Kanemaki MT, Kaito A, Wang S, Gabazza EC, Inagaki M, et al. Chk1-mediated Cdc25A degradation as a critical mechanism for normal cell cycle progression. J Cell Sci 2019;132.
4. Kohso A, Inaba H, Kanemaki MT, Gabazza EC, Toyoda H, Hirayama M, Goto H. Aurora-A Promotes Cell-Cycle Progression From Quiescence Through Primary Cilia Disassembly. Cancer Sci 2025;116:2763-2773.
5. Slenter DN, Kutmon M, Hanspers K, Riutta A, Windsor J, Nunes N, Mélius J, et al. WikiPathways: a multifaceted pathway database bridging metabolomics to other omics research. Nucleic Acids Res 2018;46:D661-d667.
6. Liberzon A, Birger C, Thorvaldsdóttir H, Ghandi M, Mesirov JP, Tamayo P. The Molecular Signatures Database (MSigDB) hallmark gene set collection. Cell Syst 2015;1:417-425.
7. Montal R, Sia D, Montironi C, Leow WQ, Esteban-Fabró R, Pinyol R, Torres-Martin M, et al. Molecular classification and therapeutic targets in extrahepatic cholangiocarcinoma. J Hepatol 2020;73:315-327.
8. Fujiwara N, Kobayashi M, Fobar AJ, Hoshida A, Marquez CA, Koneru B, Panda G, et al. A blood-based prognostic liver secretome signature and long-term hepatocellular carcinoma risk in advanced liver fibrosis. Med 2021;2:836-850.e810.

Table S1. List of real-time PCR primers

Gene	Forward Primers	Reverse Primers
	Human	
FADS1	CTGTCGGTCTTCAGCACCTCAA	CTGGGTCTTTGCGGAAGCAGTT
LPCAT3	TGGGCCGCACCATCAC	AGTTGCCGGTGGCAGTGTA
GPX4	TGGGAAATGCCATCAAGTGG	GGTCCTTCTCTATCACCAGGGG
B2M	CCACTGAAAAAGATGAGTATGCCT	CCAATCCAAATGCGGCATCTTCA
	Mouse	
Fads1	CCAGCTTTGAACCCACCAA	CATGAGGCCCATTCGCTCTA
Lpcat3	GGCCTCTCAATTGCTTATTTCA	AGCACGACACATAGCAAGGA
Gpx4	CCTCTGCTGCAAGAGCCTCCC	CTTATCCAGGCAGACCATGTGC
B2m	CTGCTACGTAACACAGTTCCAC	CATGATGCTTGATCACATGTCT

Supplementary Figure 1

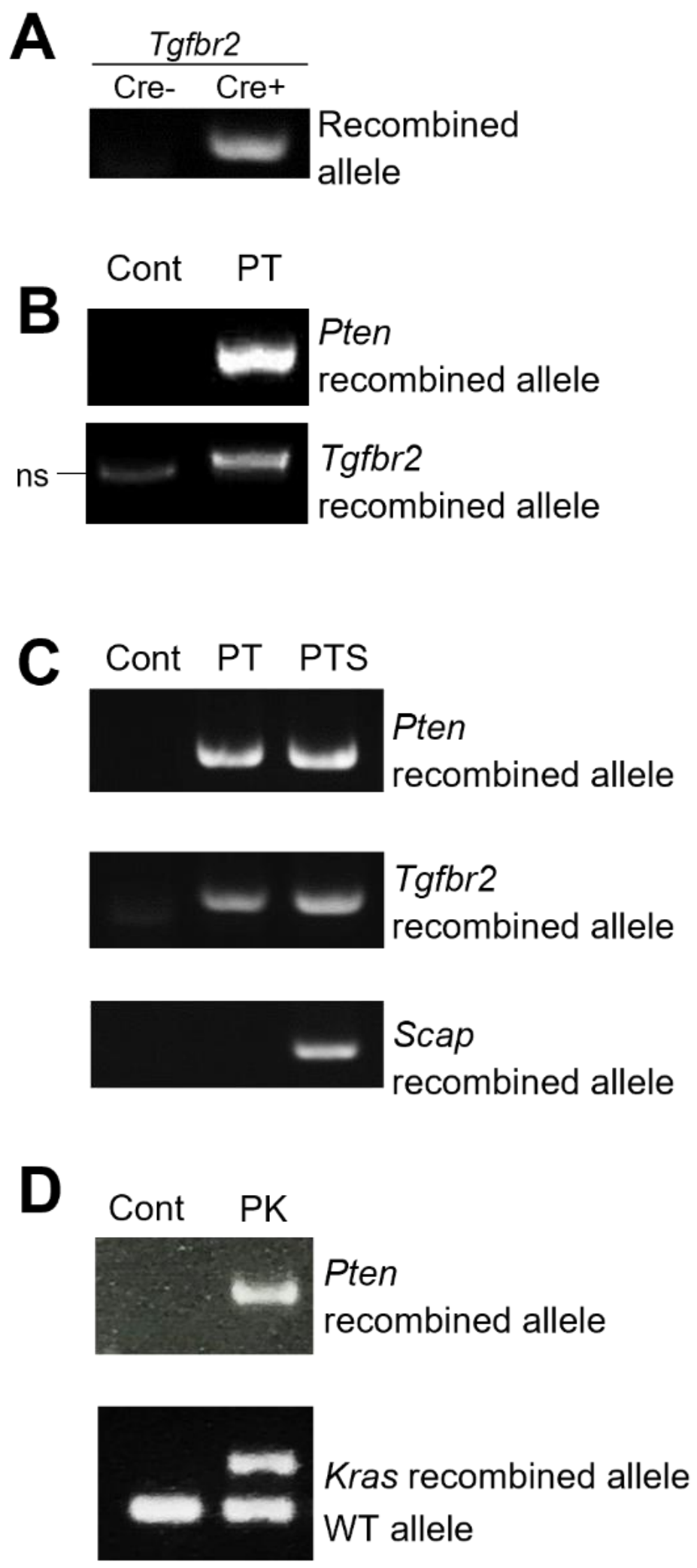

Fig. S1. Confirmation of gene recombination induced by Cre-expressing lentivirus in organoids. Gene recombination in TGFβR2-KO organoids (A), PT organoids (B), PT and PTS organoids (C), PK organoids (D) was confirmed by PCR.

Supplementary Figure 2

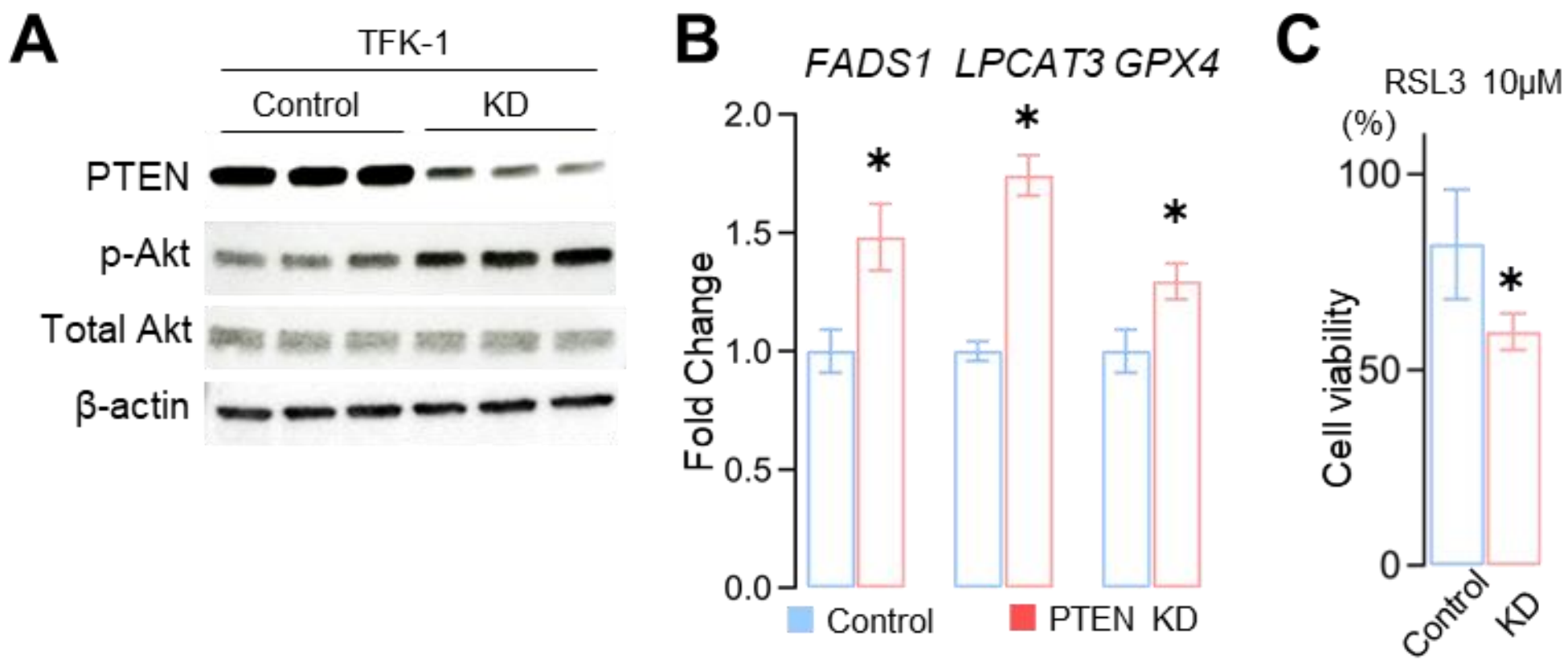

Fig. S2. Effects of PTEN knockdown in the human eCCA cell line TFK-1. (A) Knockdown of PTEN and increased Akt phosphorylation were confirmed by western blotting 48 h after transfection with control or PTEN siRNA. (B) Expression levels of the indicated genes in control and PTEN knockdown cells (n = 3 per group). (C) Control and PTEN knockdown cells were treated with RSL3 for 24 h, and cell viability was assessed (n = 3 per group).
