# Supplementary material for: SREBP-driven lipogenesis coupled with ferroptosis resistance as a therapeutic target in PTEN-loss extrahepatic cholangiocarcinoma
Source: JHEP Rep. 2026 Aug 29;8(11):102015. doi: 10.1016/j.jhepr.2026.102015 (PMC13693315; doi:10.1016/j.jhepr.2026.102015)
Supplement: Supplementary file 4 [file mmc2.docx]

**JHEP Reports**

**CTAT methods**

Tables for a “Complete, Transparent, Accurate and Timely account” (CTAT) are now mandatory for all revised submissions. The aim is to enhance the reproducibility of methods.

- Only include the parts relevant to your study
- Refer to the CTAT in the main text as ‘Supplementary CTAT Table’
- Do not add subheadings
- Add as many rows as needed to include all information
- Only include one item per row

**If the CTAT form is not relevant to your study, please outline the reasons why:**

1.1 **Antibodies**

| **Name** | **Citation** | **Supplier** | **Cat no.** | **Clone no.** |
| --- | --- | --- | --- | --- |
| Ki-67 |  | Genetex | GTX16667 |  |
| Phospho-Akt |  | Cell Signaling Technology | 4060 |  |
| α-SMA |  | Dako | M0857 |  |
| F4/80 |  | Bio-Rad | MCA497 |  |
| CD45 |  | eBioscience | 14-0451-82 |  |
| CK19 (IF) |  | Santa Cruz | SC-33111 |  |
| p21 |  | Abcam | 107099 |  |
| SREBP1 |  | Merck Millipore | MABS1987 |  |
| CK19 (DAB) |  | Merck Millipore | MABT913 |  |
| Phospho-ERK |  | Cell Signaling Technology | 4370 |  |
| Spliced-XBP1 |  | Cell Signaling Technology | 12782S |  |
| p65 |  | Cell Signaling Technology | 8242 |  |
| 4-HNE |  | JaICA | MHN-100P |  |
| PTEN (WB) |  | Cell Signaling Technology | 9559 |  |
| Phospho-Akt (WB) |  | Cell Signaling Technology | 4060 |  |
| Akt (WB) |  | Cell Signaling Technology | 4691 |  |
| Beta-Actin(WB) |  | BioVision | #3598-100 |  |
|  |  |  |  |  |

1.2 **Cell lines**

| **Name** | **Citation** | **Supplier** | **Cat no.** | **Passage no.** | **Authentication test method** |
| --- | --- | --- | --- | --- | --- |
| NOZ |  | JCRB Cell Bank | JCRB1033 | Under 10 | STR profiling |
| TGBC-14 |  | RIKEN BRC | RCB1186 | Under 10 | STR profiling |
| TFK-1 |  | RIKEN BRC | RCB2537 | Under 10 | STR profiling |

1.3 **Organisms**

| **Name** | **Citation** | **Supplier** | **Strain** | **Sex** | **Age** | **Overall n number** |
| --- | --- | --- | --- | --- | --- | --- |
| *P-CK19-Cre^ERT^* | References [18],[21] | See Material and Method | C57BL/6 | Male /Female | 7-8 weeks | 36 |
| *PT-CK19-Cre^ERT^* | References  [18],[19],[21] | See Material and Method | C57BL/6 | Male /Female | 7-8 weeks | 45 |
| *PT-LacZ-CK19-Cre^ERT^* | References  [18],[19],[20], [21] | See Material and Method | C57BL/6 | Male /Female | 7-8 weeks | 20 |
| *PK-CK19-Cre^ERT^* | References [18],[21] | See Material and Method | C57BL/6 | Male /Female | 7-8 weeks | 45 |
| *PTS-CK19-Cre^ERT^* |  | See Material and Method | C57BL/6 | Male /Female | 7-8 weeks | 10 |
| *PKS-CK19-Cre^ERT^* |  | See Material and Method | C57BL/6 | Male /Female | 7-8 weeks | 10 |
| *PK-Alb* | Reference [8] | See Material and Method | C57BL/6 | Male /Female | 4-5 weeks | 10 |
| *PKS-Alb* |  | See Material and Method | C57BL/6 | Male /Female | 4-5 weeks | 8 |
| *Tgfbr2^flox/flox^* (Organoid) | Reference [19] | See Material and Method | C57BL/6 | Male /Female | 6 weeks | 3 |
| *Pten^flox/flox^* /*Tgfbr2^flox/flox^* (Organoid) |  | See Material and Method | C57BL/6 | Male /Female | 6 weeks | 3 |
| *Pten^flox/flox^* /*Tgfbr2^flox/flox^ /Scap^flox/flox^* (Organoid) |  | See Material and Method | C57BL/6 | Male /Female | 6 weeks | 3 |
| *Pten^flox/flox^* /*Kras^G12D^* (Organoid) |  | See Material and Method | C57BL/6 | Male /Female | 6 weeks | 3 |
| WT |  | CLEA Japan | C57BL/6 | Male /Female | 11-12 weeks | 10 |

1.4 **Sequence based reagents**

| **Name** | **Sequence** | **Supplier** |
| --- | --- | --- |
| Human *FADS1* F/R | See Supplementary Table 1 | FASMAC |
| Human *LPCAT3* F/R | See Supplementary Table 1 | FASMAC |
| Human *GPX4* F/R | See Supplementary Table 1 | FASMAC |
| Human *B2M* F/R | See Supplementary Table 1 | FASMAC |
| Mouse *Fads1* F/R | See Supplementary Table 1 | FASMAC |
| Mouse *Lpcat3* F/R | See Supplementary Table 1 | FASMAC |
| Mouse *Gpx4* F/R | See Supplementary Table 1 | FASMAC |
| Mouse *B2m* F/R | See Supplementary Table 1 | FASMAC |
| Human *PTEN*-specific gRNA | See Supplementary Materials and Methods | FASMAC |
| SiRNA *PTEN* |  | Santa cruz(sc-29459) |
| SiRNA Control |  | Santa cruz(sc-37007) |

1.5 **Biological samples**

| **Description** | **Source** | **Identifier** |
| --- | --- | --- |
| N/A |  |  |

1.6 **Deposited data**

| **Name of repository** | **Identifier** | **Link** |
| --- | --- | --- |
| NCBI Gene Expression Omnibus database | GSE132305 | www.ncbi.nlm.nih.gov/geo |

1.7 **Software**

| **Software name** | **Manufacturer** | **Version** |
| --- | --- | --- |
| GraphPad Prism | GraphPad | 10.0 |
| R statistical software | The R Foundation |  |
| Adobe Photoshop | Adobe | V27.8 |
| STAR | PMID: 23104886 |  |
| DESeq2 | PMID: 25516281 |  |
| Morpheus | Broad Institute |  |
| Leica Application Suite X | Leica Microsystems | - |

1.8 **Other (*e.g*. drugs, proteins, vectors etc.)**

| **Name** | **Supplier** | **Cat no. / ID** |
| --- | --- | --- |
| Tamoxifen | Sigma-Aldrich | T5648-5G |
| Recombinant TGF-beta 1 | Peprotech | 100-21C |
| RSL3 | Selleck | S8155 |
| KK-052 | See reference [34] | - |
| RNAscope^TM^ probe Mm-Tgfb1 | Advanced Cell Diagnostics | 407751 |
| pSpCas9(BB)-2A-Puro V2.0 (pX459) | Addgene DOCX | 62988 |
| pMK293 vector | Addgene DOCX | 72831 |
| Viafect | Promega | E4981 |
| Lipid Peroxidation Probe BODIPY 581/591 C11 | Dojindo | 344-10061 |
| TBARS Assay Kit | Cayman Chemical | 10009055 |
| GFP-Puro (CAG Promoter) lentivirus | GenTarget Inc | LPV579 |
| CRE (GFP-Puro) (CAG Promoter) lentivirus | GenTarget Inc | LPV576 |

1.9 **Please provide the details of the corresponding methods author for the manuscript:**

Hayato Nakagawa

Department of Gastroenterology and Hepatology, Mie University Graduate School of Medicine,

2-174 Edobashi, Tsu-shi, Mie-ken, Japan, 514-8507

E-mail: nakagawah@med.mie-u.ac.jp

Tel: +81-59-231-9238; Fax: +81-59-231-5269

**2.0	Please confirm for randomised controlled trials all versions of the clinical protocol are included in the submission. These will be published online as supplementary information.**

**N/A**
